# Morphological and metabarcoding dietary analysis of the cunner wrasse ( *Tautogolabrus adspersus* ) revealed significant regional variation, with large overlap between its common prey species and biofouling animals living on salmonid sea cages

**DOI:** 10.1111/jfb.70013

**Published:** 2025-03-04

**Authors:** Christopher J. D. Bender, Camden D. Moir, Mehrdad Hajibabaei, Elizabeth G. Boulding

**Affiliations:** ^1^ Department of Integrative Biology University of Guelph Guelph Ontario Canada; ^2^ Centre for Biodiversity Genomics University of Guelph Guelph Ontario Canada

**Keywords:** cleaner fish, diet, feeding ecology, metabarcoding, sustainable aquaculture, wild populations

## Abstract

The stomach‐less cunner wrasse (*Tautogolabrus adspersus*) has been experimentally used as a biological control agent for salmon lice that infest Atlantic salmon (*Salmo salar*) and to remove biofouling inside sea cages. The cunner demonstrates a strong population structure, suggesting that its diet, and therefore its usefulness for biological control, could differ among its populations along 1086 km of eastern Canada, in response to the biogeography of its prey species. Gastrointestinal tract samples were collected across 14 locations throughout five distinct regions from Southern Nova Scotia to Eastern Newfoundland between 2018 and 2022. Primary constituents of diet, identified using morphology and by percentage weight, were mussels, bryozoans, ascidians, gastropods, unidentified digested material and barnacles. Dietary DNA (dDNA) metabarcoding identified mussels in 46% of guts, amphipods in 45%, bryozoans in 31%, ascidians in 28% and sea anemones in 18%. Sea lice were rare yet present in samples from three separate regions. Permutational multivariate analysis of variance (PERMANOVA) based on DNA metabarcoding suggested that sampling region, location and year all significantly influence diet composition. Regional divergence in diet was greatest between Southwestern Nova Scotia and Northeastern Newfoundland. Invasive cionid ascidians were present almost exclusively in Nova Scotian samples, whereas brittle stars were present almost exclusively in Northeastern Newfoundland samples. dDNA metabarcoding enabled the detection of soft‐bodied prey and often identified prey to the species level. Cunner were demersal feeders on neritic sessile or slow‐moving benthic animals that comprise the biofouling community. In addition to preying on sea lice and invasive ascidians, we predict that cunner wrasses will reduce the density of biofouling communities on structures used in marine aquaculture.

## 1 INTRODUCTION

The aquaculture industry has seen significant levels of growth over the past four decades, with biomass production of animals multiplying by over nine times in quantity from 10 million tons in 1987 to over 94.4 million tons by 2022 (FAO, 2024; Naylor et al., 2021; Tveterås et al., 2012). These recent metrics of production represent the first time in human history wherein the production of biomass from aquaculture practices surpassed that of wild catch (FAO, 2024). The continued success of culturing of salmonid fishes in sea cages, an integral taxon of this industry, will depend on solutions to parasitism, disease and the propagation of biofouling communities. Biofouling, defined as the propagation and growth of non‐target organisms across hard surfaces around and within sea cages, has continued to be one of the major sources of pest infestation of aquaculture facilities for decades (Fitridge et al., 2012; Hodson et al., 1997). The growth of non‐target organisms creates problems concerning the development, survival and risk of disease in target stock by preventing the bidirectional flow of water, oxygen and nutrients throughout the pen (Amara et al., 2018; Bi et al., 2018; Fitridge et al., 2012). Various methods of net‐cleaning are available, yet these solutions present issues surrounding extensive labour and investment costs regarding physical remediations, whereas the accumulation of harmful biocides and heavy metals in the local environment are prominent criticisms of chemical solutions (Bannister et al., 2019; Fitridge et al., 2012; Tornero & Hanke, 2016). There remains a need for the development of biofouling control methods that are environmentally sustainable and cost‐effective.

The use of cleaner wrasses in salmonid sea cages in regions of Europe with warmer sea temperatures (Cavrois‐Rogacki et al., 2019; Geitung et al., 2020; Yuen et al., 2019) for the management of sea lice has been successful in maintaining parasite loads well below the legal limits (Blanco Gonzalez & Boer, 2017; Brooker et al., 2018, 2020; Sistiaga et al., 2021). In European aquaculture, both the lumpfish (*Cyclopterus lumpus*) and several local wrasse (Labridae) species are used in salmonid sea cages, including the ballan (*Labrus bergylta*), goldsinny (*Ctenolabrus rupestris*) and corkwing (*Symphodus melops*) wrasses (Erkinharju et al., 2021). Along the coast of Atlantic Canada and the northwestern United States, the cunner (or blue perch), *Tautogolabrus adspersus* (Walbaum, 1792) is the only neritic wrasse species present that is suitable as a cleaner fish (Boyce et al., 2018; Costa et al., 2016). Although cold‐water‐dependent lumpfish from the Northwest Atlantic have also been used for biocontrol of sea lice in local salmonid sea cages, increasing sea water temperatures have increased their mortality rates; this has created a need for an alternative cleaner fish like the cunner wrasse, which can physiologically tolerate warmer temperatures (Boyce et al., 2018; Hvas et al., 2018; Reeve et al., 2022). Cunners engage directly in cleaner‐client interactions with lice‐parasitized salmonids in laboratory settings, providing further evidence of this wrasse species' potential for use as a cleaner fish (Costa et al., 2016; Mackinnon, 1995; Whittaker et al., 2021).

*T. adspersus* represents the northernmost member of the Labridae (wrasses) family along the western Atlantic, sharing parts of its range with other tropical and subtropical wrasse species (Dew, 1976). *T. adspersus* is a broadcast‐spawning species with a planktonic larval stage but demonstrates a tendency for significant adult philopatry that limits dispersal and results in marked population differentiation based on geographic isolation (Nugent et al., 2023; Pottle & Green, 1979; Serchuk & Cole, 1974). *T. adspersus* inhabits sheltered rocky nearshore and estuarine environments with high substrate heterogeneity (Green & Farwell, 1971; Pottle & Green, 1979; Serchuk & Cole, 1974). A typically abundant species that is frequently found in large congregations of 10–50 individuals, *T. adspersus'* distribution range spans from Chesapeake Bay in Virginia to the Labrador Straight north of Newfoundland (Green et al., 1984; Green & Farwell, 1971; Kelly et al., 2014; Pottle & Green, 1979; Serchuk & Cole, 1974). The presence of significant population genetic structuring across such a vast geographic range (Nugent et al., 2023) presents opportunities for adaptation to local environmental conditions and communities, potentially altering prey consumption tendencies between geographically isolated populations.

Limited information about the natural diet of *T. adspersus* has been recorded, and all previous studies used traditional morphological methods while focusing on a single region. An early morphological study performed in New York classified *T. adspersus* as an opportunistic generalist feeder, targeting benthic organisms like mussels and isopods, while occasionally consuming other invertebrates found throughout the water column (Olla et al., 1975). Given the wide geographic range over which this species resides naturally (Dew, 1976), and its high level of population genetic structure (Nugent et al., 2023), there exists great potential for regional dietary variation in response to changes in prey availability with latitude. The lack of scientific literature in the past 30 years on the topic of *T. adspersus* natural prey consumption, as well as a lack of studies observing *T. adspersus* diet across multiple geographic areas, has highlighted a need for a rigorous and contemporary analysis of the diet of this species. This information could be especially useful for determining the potential of this species as a cleaner fish option, given there is significant overlap between frequent prey items in the natural diet of *T. adspersus* and the commonly observed species in biofouling/pest communities in aquaculture.

Traditional forms of gut content analysis have relied on the visual identification of prey items to specific taxa, where quantification of a taxon is determined as the ratio of its weight relative to the weight of the entire gut sample (Carreon‐Martinez et al., 2011; O'Dell et al., 2020). This method, although cost effective and semi‐quantitative, has inherent flaws and biases that arise from (1) difficulties in morphological identification of well‐digested prey to a specific taxon, (2) comparison of abundances of taxa digested at different rates, (3) time biases of when the sample is taken relative to when it is dissected and (4) inherent digestive biases resulting from harder body parts (i.e., exoskeletons) being digested slower and thus being more likely to be identified to the correct taxon compared to soft tissues (Carreon‐Martinez et al., 2011; O'Dell et al., 2020; Symondson, 2002). To overcome these disadvantages, researchers have implemented DNA sequencing of amplicons using universal PCR primers to assess organismal dietary ecology, which is effective even when the absolute abundance of DNA from consumed prey is low (Carreon‐Martinez & Heath, 2010; Symondson, 2002).

Dietary DNA (dDNA) metabarcoding of stomach contents has emerged as a tool for assessing dietary composition of species, with higher levels of taxonomic resolution when compared to traditional methods of diet analysis (Berry et al., 2015; Canals et al., 2024; Harms‐Tuohy et al., 2016; Jakubavičiute et al., 2017; Nalley et al., 2022). The use of mitochondrial cytochrome oxidase subunit I (COI) fragment sequences as an identifier of taxonomic groupings has revolutionized the biodiversity analysis of animals (Bucklin et al., 2021; Hebert et al., 2003; Ratnasingham & Hebert, 2007; Taberlet et al., 2012). DNA metabarcoding using next‐generation sequencing of bulk samples (Elbrecht et al., 2019; Gibson et al., 2014; Porter et al., 2019), the continued expansion and development of barcode databases in recent years (Ratnasingham & Hebert, 2007; Ratnasingham & Hebert, 2013) and modern bioinformatic methods for identifying animals in freshwater benthic samples (Hajibabaei et al., 2012; Robinson et al., 2022) have resulted in the successful integration of these techniques in dietary analysis of fishes (Bourlat et al., 2021; Jakubavičiute et al., 2017; Roy & Boulding, 2024; Schaafsma et al., 2024).

Prior to the integration of *T. adspersus* as a cleaner fish in sea cage environments, a robust understanding of the species' natural dietary composition needed to be developed with a focus on the prevalence of common biofouling and pest invertebrate species. Only a joint morphological and metabarcoding approach to dietary determination can provide an accurate and in‐depth view of the natural diet of this species across its broad geographic range and accurately identify which pest species relevant to aquaculture are being consumed within a given region. To address this gap in the literature, traditional morphological and modern dDNA approaches for dietary analysis of *T. adspersus* collected at 14 different sites throughout Atlantic Canada were used to examine differences in dietary composition across 1000 km of its geographic range. An initial investigation suggested that there were large differences in dietary composition between a single site in Hermitage Bay in Southern Newfoundland and all Nova Scotian regions that were sampled (Moir, 2021). This led to the development of the hypothesis that biogeographic variation in prey taxa and geographical separation imparted by the Gulf of St. Lawrence would result in differences in the makeup of diets for individuals from different regions, with larger differences being observable between specimens captured north (Newfoundland) and south (Nova Scotia) of the Gulf of St. Lawrence.

## 2 MATERIALS AND METHODS

### 2.1 Study region and cunner GI tract collection/processing

**FIGURE 1:**
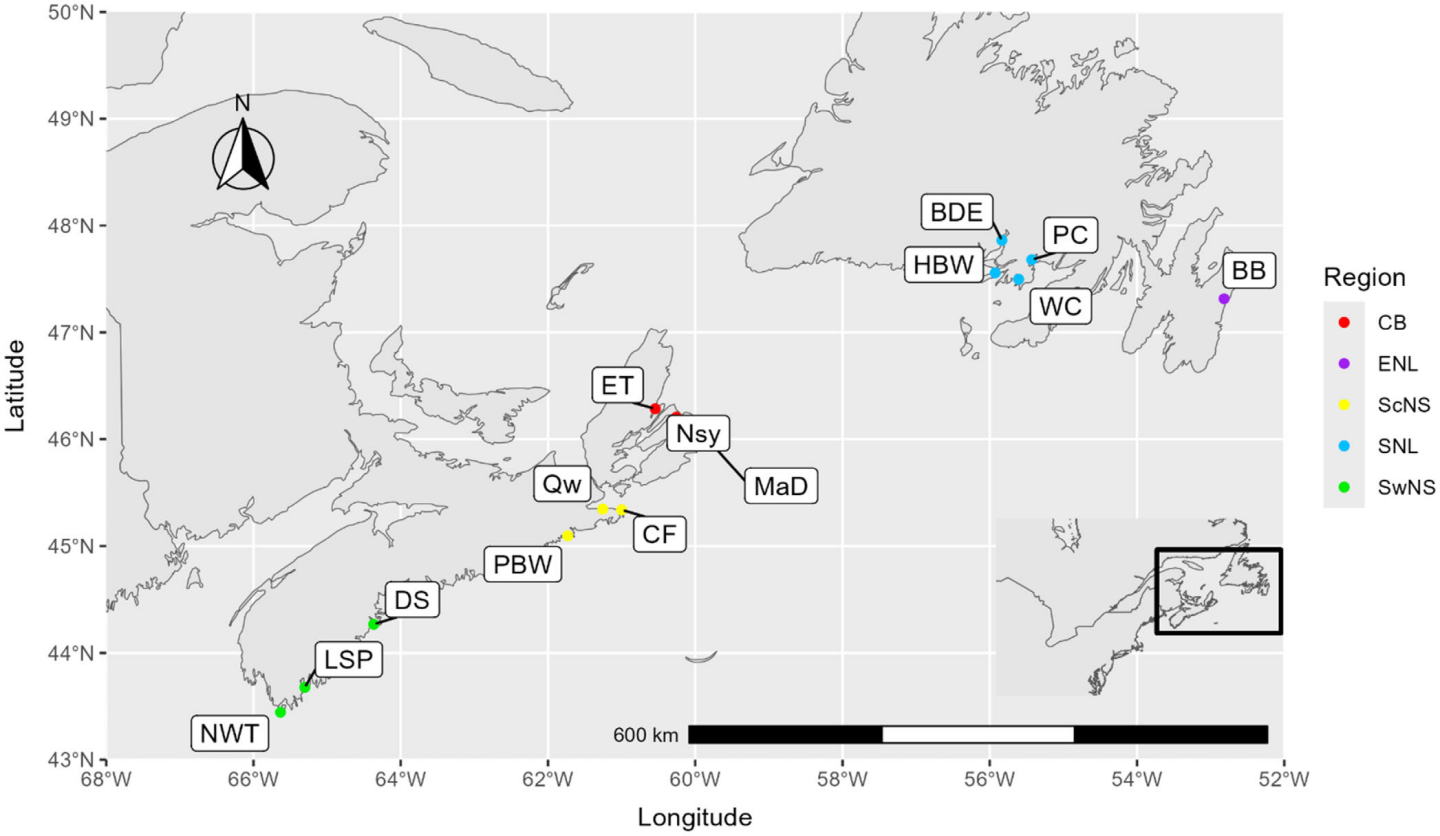
Study sample site of all 14 locations throughout Atlantic Canada. Sample regions and their respective abbreviations were denoted as: Southwest Nova Scotia (SwNS, 44° N, 65° W; *N* = 30), Southcentral Nova Scotia (ScNS, 45° N, 61° W; *N* = 30), Cape Breton (CB, 46° N, 60° N; *N* = 30), Southern Newfoundland (SNL, 47.5° N, 56° W; *N* = 90) and Eastern Newfoundland (ENL, 47° N, 53° W; *N* = 25). Cape Breton was represented by (1) Main‐a‐dieu (MaD), (2) Englishtown (ET) and (3) North Sydney (NSy). Southwestern Nova Scotia was represented by (1) Newellton (NWT), (2) Lower Sandy Point (LSP) and (3) Dublin Shore Wharf (DSW). Southcentral Nova Scotia was represented by (1) Canso Fisherman's Wharf (CF), (2) Queensport (QP) and (3) Port Bickerton West (PBW). Southern Newfoundland was represented by four sample sites in (1) Hermitage Bay Wharf (HBW), (2) Bay D'Espoir (BDE), (3) Wreck Cove (WC) and (4) Pool's Cove (PC), whereas Eastern Newfoundland was represented by a single sample site at Bay Bulls (BB). A singular site (HBW) was sampled in two separate sample years (2019 and 2022).

*T. adspersus* (*N* = 205) were obtained from 14 coastal bays situated across a 1086‐km stretch of the Northwest Atlantic coastline, ranging from Cape Sable Island in Nova Scotia to Bay Bulls in Eastern Newfoundland (Figure 1). These 14 locations were grouped into five distinct regions along the Atlantic Canadian coast, with groupings based on latitude and proximity to other sampling sites (Figure 1). Sampling took place over the course of 3 years, with 20 Hermitage Bay samples being collected in 2018, all of the Nova Scotian samples being collected in 2019 and the remaining Southern Newfoundland and Eastern Newfoundland samples collected in 2022. All samples were collected in early‐to‐mid August of their respective year. All samples were collected through targeted fishing techniques (single‐line angling, modified minnow traps) from wharves/docks/jetties at depths ranging from 2 – 10 meters with benthic substrates primarily composed of cobble/rocky to gravel bottoms. All bait used was from either terrestrial meat sources (i.e., chicken, beef, pork, turkey) or herring bait so that bait‐specific sequences could be removed from the metabarcoding datasets.

*T. adspersus* collected for this study were adults, ranging in size from 89 to 312 mm in total length [*μ* = 196.82 mm, standard error (SE) ± 0.24 mm] and between 11 and 412 g in total weight (*μ* = 141.57 g, SE ± 0.49 g). To test for potentially confounding effects that differences in body size may have on dietary composition, a Kruskal‐Wallis test and a subsequent post‐hoc Dunn's test for variance was conducted on length and weight measurements between sampling regions. The results of the Kruskal‐Wallis test revealed statistically significant effects of region on specimen total length and total weight, potentially biasing any obtained results (Figure S2). The results of Dunn's test revealed that, although certain pairs of regions did significantly differ in terms of weight and size, there was no singular region that always had a different range of sizes when all pair‐wise comparisons were analysed, suggesting that although size differences may influence dietary results, the overall effect of this confounding variable is likely muted (Table S1).

Weight measurements of whole GI tracts, total digestive content and each diet category were recorded using an analytical balance (±0.01 g) after the thawing of samples from freezing temperatures. All weights presented represent the wet weight at the time of measurement. Morphological assessment of diet contents involved the separation of macroscopic prey items from digested tissue clumps. Undigested, intact prey items were identified to the lowest possible taxonomic level using a Wild dissecting microscope from Leica Microsystems (Leica Microsystems, 2024). Unidentifiable organic matter [classified as Other (Organic)] was recorded from the remaining gut contents. Three samples from Hermitage Bay (2022), one sample from Lower Sandy Point (2019) and two samples from Port Bickerton West (2019) were empty of contents and were removed from the study. This left a total of 107 (2018/2019) samples and 92 (2022) samples (*N* = 199) containing sufficient contents for diet analysis. After morphological identification, all of the digested gut contents from each sample were dried (for DNA extraction purposes) at room temperature (21°C) until all preservative ethanol had evaporated (roughly 2–6 h), and contents were homogenized using a liquid nitrogen‐cooled, sterile mortar and pestle. Resulting homogenates from this mechanical shearing process that contained more than 220 mg were divided into replicates of 180–220 mg using a Mettler balance (±0.001 g) (Mettler Toledo, 2024). Sample homogenates (a single replicate per sample, chosen randomly) were mechanically digested further using a Thermo Savant FastPrep FP120 homogenizer with liquid nitrogen and a ceramic bead that was roughly 1 cm in diameter (Thermo Savant, 2001). The homogenization and DNA extraction process was completed following established lab protocols for DNA extraction of gut contents (Roy & Boulding, 2024).

Genomic DNA (gDNA) was extracted with the Qiagen QIAamp Fast DNA Stool Mini kit (Qiagen, 2024) following the manufacturer's protocol with two exceptions: (1) a prolonged period of digestion in an increased concentration of Proteinase K solution (2–5 h on a shaker plate inside a 56°C oven, depending on the tissue density contained within the samples and the concentration of Proteinase K of either 100–160 μL), and (2) a reduction in final elution volume (50 mL) to further concentrate the purified DNA. Extracted gDNA was stored at −20°C prior to fluorometric quantitation with Qubit 2.0 Fluorometer (Thermo Fisher Scientific, 2024a) using the Invitrogen dsDNA BR Assay Kit (available at: https://www.thermofisher.com/order/catalog/product/Q32850) at the AAC Genomics facility within the University of Guelph (AAC, 2024). Five negative controls were included during the extraction process to assess for potential DNA contamination, with all returning near‐zero (<0.001 ng/μL) levels of detected DNA during Qubit fluorometry. DNA extractions and polymerase chain reaction (PCR) amplification were successful for 195 (2018/2019: *N* = 107, 2022: *N* = 88) samples.

### 2.2 PCR amplification and DNA metabarcoding

PCR amplification of genomic DNA was completed by the Hajibabaei Lab in the Center for Biodiversity Genomics (CBG) at the Biodiversity Institute of Ontario (CBG, 2024). A total of three published primer sets were chosen to amplify the segments of the mitochondrial COI ‘barcoding’ region: (1) B_F/ArR5_R (BR5), (2) LCO1490_F/230_R (F230R) and (3) mlCOIint_F/jgHCO2198_R (ml‐jg) (Porter & Hajibabaei, 2018). Primers were chosen based on either their specificity for marine and terrestrial invertebrates (BR5, ml‐jg), the overall depth of sequence capture for various arthropods (BR5, F230R), shorter amplicon lengths (F230R) or based on their proven successes with digested prey material (ml‐jg; Harms‐Tuohy et al., 2016). Negative controls without template DNA for each PCR amplification were utilized to validate sample amplification. The subsequent amplicons from each PCR library were purified using MiniElute PCR purification columns (Qiagen) and eluted with 30 μL of molecular grade water. The generated amplicon libraries were tested for quality following each PCR step via 1.5% agarose gel electrophoresis. Two‐step PCR protocols were used for initial amplification with COI primers, followed by a second round of amplification using modified COI primers containing Illumina tailed adapters (Robinson et al., 2022). Libraries were prepared to a final volume of 25 μL as a mix of the following components: 2 μL of DNA template, 0.5 μL of forward (10 mM) and 0.5 μL of reverse primer (10 mM), 0.5 μL of dNTPs (10 mM), 2.5 μL of 10× reaction buffer (200 mM Tris–HCl and 500 mM KCl, pH 8.4), 1 μL of MgCl_2_ (50 mM), 0.5 μL of Platinum Taq Polymerase (5 U/μL) and 17.5 μL of molecular‐grade H_2_O. The PCR thermocycling regime during rounds of amplification of COI fragments using the BR5 and F230R primers followed an established lab protocol: (1) process initialization at 95°C for 5 min using a heated lid, (2) denaturation at 94°C for 40 s, (3) fragment annealing at 46°C for 1 min, (4) initial extension at 72°C for 30 s repeated for 30 cycles and (5) a final extension period of 5 min at 72°C, following which PCR products were held at 4°C. A modified regime was used specifically for COI amplification using the ml‐jg primer: (1) process initialization at 95°C for 1 min using a heated lid, (2) denaturation at 94°C for 15 s, (3) fragment annealing at 46°C for 15 s, (4) initial extension cycle at 72°C for 10 s that was repeated 30 times and (5) a final extension cycle at 72°C for 1 min, following which PCR products were held at 4°C. Dual indexing of all generated amplicons was completed using Illumina's Nextera Indexes (Illumina, 2024; Product Ref: FC‐121‐1011) in a third PCR process for 12 cycles to include sample identifiers, then pooled into a microcentrifuge tube. The resulting pooled libraries were purified with AMpure magnetic beads, then quantified on a TBS‐380 Mini‐Fluorometer (Turner BioSystems, 2007) using a Quant‐iT PicoGreen dsDNA assay (Thermo Fisher Scientific, 2024b; product ref.: P11496). Fragments for the library were assessed for average length using the Agilent DNA 7500 assay chip on an Agilent Bioanalyzer 2100 (Agilent, 2024).

Sequencing of the pooled, indexed library was completed using the Illumina MiSeq platform following the manufacturer's protocols. A single run using the MiSeq v3 sequencing kit (2 × 300 bp; FC‐131‐1002 and MS‐102‐2003) was performed with a 5% PhiX spike‐in, producing 2 × 300 bp paired‐end sequences. A total of 105 samples from 2018/2019 and 86 samples from 2022 contained raw sequences for downstream analysis (*N* = 191). Sequencing resulted in up to 62.76 million total reads and roughly 328,618 forward and reverse reads per sample before pairing. After pairing, total reads equated to roughly 29.39 million, and each sample had an average of 153,971 reads. For the three utilized primer sets and 191 total samples following trimming, this ended in roughly 25.5 million total reads and roughly 133,581 reads per sample that were to be analysed. Using a singularity established by the Digital Research Alliance of Canada (DRAC, 2024) on its CEDAR servers, the generated raw sequences were processed using the MetaWorks metabarcoding pipeline v1.12 (available on https://github.com/terrimporter/MetaWorks; Porter & Hajibabaei, 2022). Specific information regarding the bioinformatic processes for taxonomic assignments and data filtration using MetaWorks can be found in DataS1. Following metabarcode assignments using MetaWorks, a total of 24 phyla, 64 classes, 240 orders, 905 families, 1644 genera and 2064 species were classified, with a total of 21,326 COI ESVs identified across all samples.

### 2.3 Statistical analyses and data visualization

The datasets used for subsequent analyses were of varying sizes resulting from filtration and sample loss discussed earlier, with the morphological dataset comprised of 199 samples (*N* = 199), and the metabarcoding data being divided into two separate datasets depending on the specific taxonomic resolution being analysed: these contained either 191 samples (*N* = 191 at or above the genus level) or 187 samples (*N* = 187 at the species level).

The diet of *T. adspersus* was assessed morphologically using the following metrics calculated from various measurements of wet weight. Principally, this included the percentage composition of diet by weight in grams (%WC) to quantify the proportion to which each identified taxa contributed to the total weight of a given sample. At the same time, the percentage frequency of occurrence (%FOO, the number of detections of a given taxa across all samples in relation to the number of possible samples, calculated as a percentage) and proportion of occurrence (POO, the proportion of detection of a given taxa across all samples in relation to the total number of all detected taxa across all samples) of each identified taxonomic group were calculated; the specific equations used to calculate each metric can be found in Deagle et al. (2019). These were calculated for all datasets (morphological and both metabarcoding) to enable direct comparisons between datasets, as these same metrics can be applied to presence/absence data generated from metabarcode assignments. Another commonly used metric for quantification of metabarcode‐specific data that were calculated was weighted POO or wPOO, which weights the frequency of observations of a given taxonomic unit against the total amount of observations of the sample it is found, and sums up these values before averaging them across all of the samples in the study (Deagle et al., 2019). This can further provide an estimation of the relative contribution specific prey items have towards the total of all prey items within the samples they are found in, with taxa that commonly dominate the samples they are found in having unusually higher wPOO metrics compared to other taxa that may share similar values in %FOO or POO, respectively.

Other metrics can only be applied to the metabarcoding data. After filtration, data were summarized based on the total percentage of either exact sequence variants or ESVs (%ESV) or total reads assigned (relative read abundance or RRA) to each major taxonomic group at a given level of resolution for the entire dataset. Both of these metrics provide valuable and different information, as ESVs and their related metrics are representative of uniquely identified sequences (i.e., each unique ESV represents a unique genetic lineage), whereas total reads and subsequently calculated metrics (i.e., RRA) are simply measures of abundance for all of the amplified genetic material. Beyond the representation of %ESV and RRA of majorly identified taxa, data were represented in the form of a regional comparison of total counts of ESVs and reads for major taxonomic groups.

We initially visualized the metabarcoding data based on ESVs and total reads across and within the sample regions, using presence/absence of each prey taxon in each cunner stomach at the genus and species levels. The genus level worked best for organisms such as bivalves of the *Mytilus* edulis complex because of its double uniparental inheritance of the mitochondrial genome (Zouros & Rodakis, 2019), and the solitary ascidians in the genus *Ciona* because of its uncertain species boundaries (Wilson et al., 2022). Raw data were filtered for bait contaminants, and bootstrap cut‐offs were applied for shared base‐pair thresholds (sBP) between the sample and the database reference at a value of either ≥0.80 (species‐level comparisons) or ≥0.30 (genus‐level comparisons) to ensure taxonomic assignment at high confidence intervals. This is in direct accordance with the recommendations provided by the creators of MetaWorks for accurate taxonomic assignment at finer resolutions (Porter & Hajibabaei, 2022). Given our goal of exploring similarities and differences in sample composition across and within sample regions and locations, we employed a multivariate approach to data analysis to determine relative similarity between all possible pair‐wise sample comparisons based on the presence/absence of taxa identified in each cunner wrasse gut. To remove the potentially misleading effects that rare taxa may have on sample comparisons, all taxa that were present in fewer than 5% of samples (equivalent to nine or fewer separate detections within the metabarcode datasets) were removed. This was guided by previous research where the threshold of occurrence required for an item to be considered as a part of the analysis was no fewer than 10 samples, or at a frequency roughly equivalent to 1% of the most common prey item (Deagle et al., 2019; Quéméré et al., 2013). These modifications further reduced the overall size of the dataset available for multivariate analysis in the case of the two metabarcoding datasets, bringing sample sizes to *N* = 188 (genus) and *N* = 183 (species), whereas the morphological dataset maintained the same sample number as before (*N* = 199). This was a direct result of these removed samples only being comprised of proportionately rare taxa, such that when these rare taxa were removed, these samples no longer possessed any detections.

Given the nature of presence/absence data and the specific ecological context we were working in, we decided to use a principal coordinate analysis (PCoA) using Sørensen's coefficient as the dissimilarity metric for data ordination to represent relative similarities between all samples, where the calculation of similarity between two samples is completed by giving double weight to shared presences compared to shared absences (Legendre & Legendre, 2012). This coefficient was used as the basis for the distance matrix that was calculated to plot the various PCoAs generated from the three major datasets analysed (Morphological, Metabarcode‐Genus and Metabarcode‐Species). Subsequent statistical analyses were conducted via PERMANOVAs and pair‐wise multivariate analyses of the generated distance matrices. This was completed to identify whether specific independent variables (Region, Location, Year) had significant effects on the overall composition of diets between samples, and to visualize where the largest differences between independent variables (Region, Location) were. All statistical analyses were performed using R, version 4.3.1 within RStudio version 12.1.402 (RStudio, 2024), with the employment of the vegdist, wcmdscale and adonis2 functions within the descriptive community analysis R package named vegan (Oksanen et al., 2024). The pair‐wise. adonis function within the R package pair‐wise. adonis was used to calculate pair‐wise multilevel analyses (Martinez Arbizu, 2020).

## 3 RESULTS

### 3.1 Morphological analysis

**FIGURE 2:**
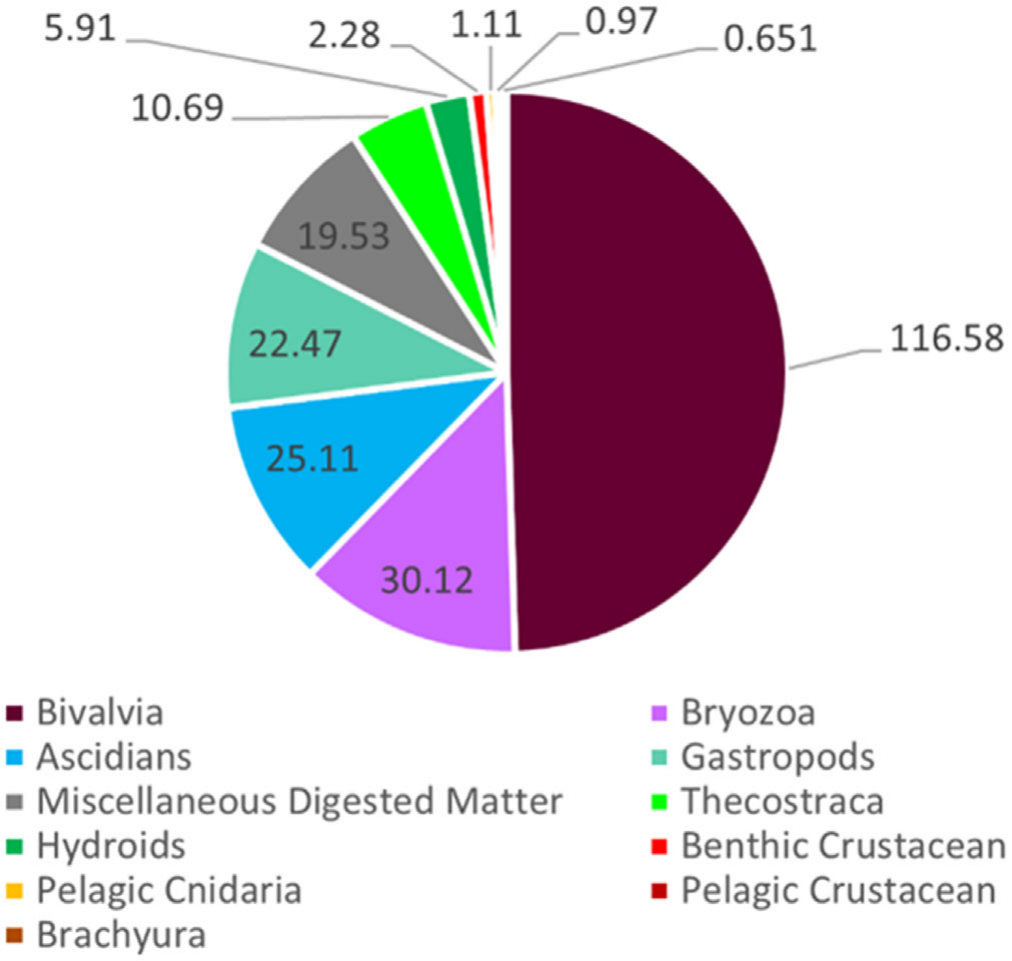
Percentage weight by composition in grams (%WC) for the major taxonomic groups identified within the morphological analysis. Listed numeric values represent total weight of each respective taxonomic group in grams, as well as the percentage that each group contributes towards the total of all wet weight measured.

The morphological analysis of gut contents revealed a total of 10 unique and distinguishable taxonomic groups, with various other identifications made to lesser degrees of confidence (and thus grouped together in the ‘Other’ category to minimize potential identification errors). Morphological analyses revealed the frequent consumption of benthic organisms, many of which are entirely sessile and significantly contribute to biofouling on hard surfaces (Figure 2). The most dominant prey taxa in terms of weight proportion (%WC) included bivalves, ascidians, bryozoans and gastropods across all of our samples, representing over 82% of the total weight recorded.

**FIGURE 3:**
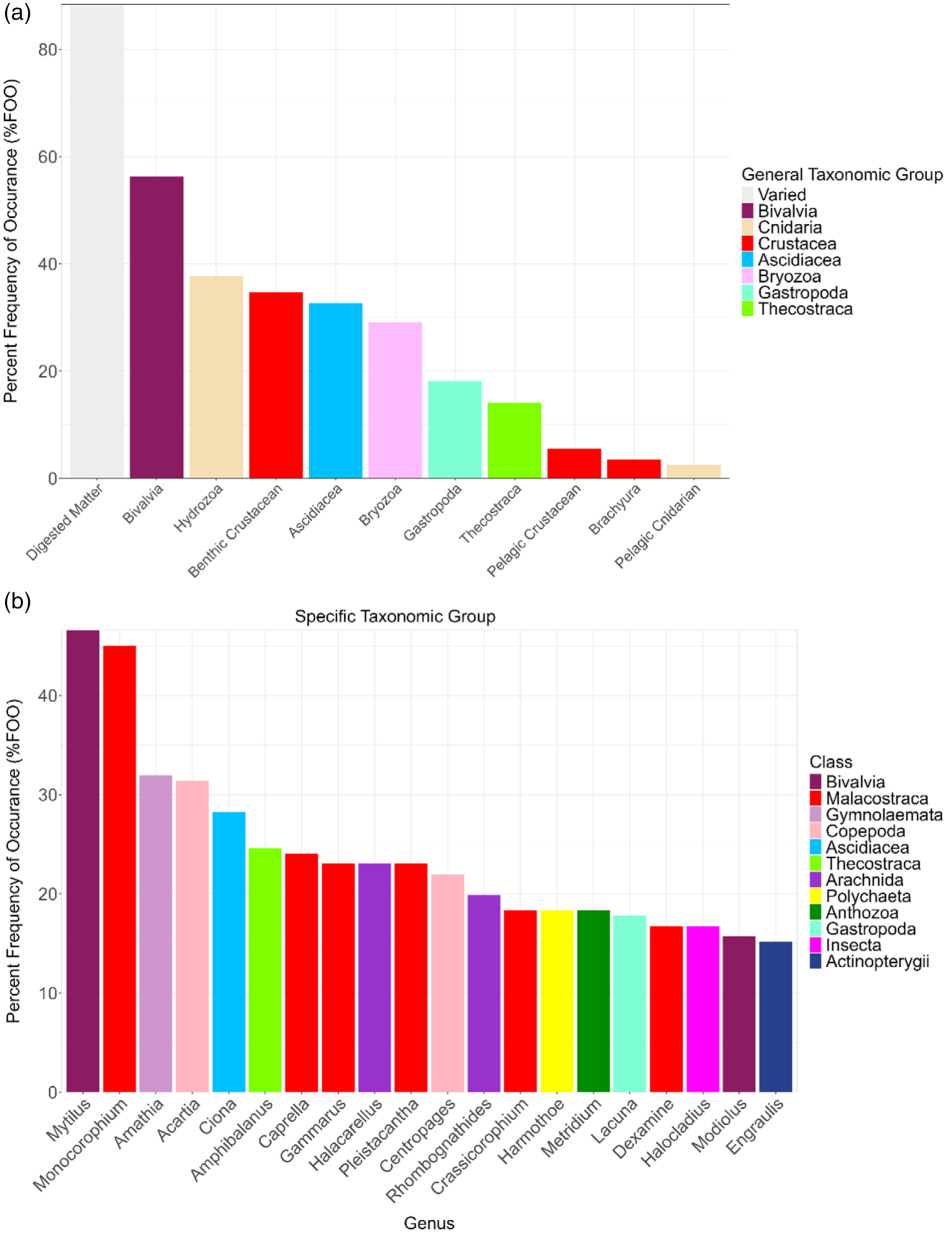
Visualizations of percentage frequency of occurrence in cunner gut samples [Percentage frequency of occurrence (%FOO)] for: morphological analysis (a) and metabarcoding to the genus level for the top 20 most‐frequently identified taxa (b).

Prey categories that were commonly identified across samples in the morphological dataset included bivalves, bryozoans, solitary ascidians and benthic crustaceans (Table S3; Figure 3a). Bivalves were found in approximately 56% of all samples when analysing %FOO, whereas other species that had large contributions to overall dietary weight, such as noted species bryozoans and ascidians, were found in over 25% of samples (%FOO). Hydroids and benthic crustaceans were identified in over 32% of all samples (%FOO) despite proportionately small contributions to overall weight consumed.

### 3.2 Metabarcoding identifications: Pre‐ and post‐filtering taxa summaries

Unfortunately, because not all eastern Canadian coastal animal species were present within the reference databases, many of these initial detections were at shared base pair levels that were well below the recommended thresholds to be considered accurate for identification using COI. At a shared base pair of 0.3, as is recommended for genus‐level analysis (gBP), we detected 231 genera which were classified as being from 14 phyla, 32 classes, 79 orders, 182 families, which resulted in 4426 COI ESVs across all samples. At a shared base pair of 0.8, as is recommended for species‐level analysis (sBP), we detected 228 species which were classified as being from 14 phyla, 30 classes, 73 orders, 162 families, 198 genera, which resulted in 3344 COI ESVs across all samples. These revised ESV totals were the subsequent basis for all further analyses of the dDNA metabarcoding data at the genus or species level, including the occurrence metrics (%FOO, POO, etc.), as well as for the methods of ordination and subsequent statistical analysis that enabled the visualization of similarities and differences between individual samples.

### 3.3 FOO: Morphological versus metabarcoding

Comparison of the shared occurrence metrics (%FOO, POO) between the morphological dataset and the metabarcoding datasets reinforced the initial observations of the morphological analysis, with diets commonly consisting of bivalves, solitary ascidians, bryozoans and benthic amphipods (Table 1; Figure 3). The %FOO analysis for the metabarcode datasets revealed a large frequency of detection of various bivalves, with the genus *Mytilus* being found in over 46% of samples, and the genus *Modiolus* identified in over 15% of samples. Various other genera of note include ascidians like *Ciona* (>28%), bryozoans like *Amathia* and *Membranipora* (>31%, >13%) and benthic crustacean genera such as *Monocorophium*, *Caprella*, *Gammarus* and *Crassicorophium* (>45%, >24%, >23%, >18%). There was a proportionately large percentage of different genera of copepods like *Acartia* and *Centropages* (>31%, >21%), as well as the presence of the salmon louse (*L. salmonis*) in three separate samples (>1.5%), which is a pest species of particular concern regarding Atlantic salmon aquaculture.

**TABLE 1:** Taxa counts, %FOO, POO and wPOO, as well as RRA for the 20 most frequently identified genera and species across the entire study for the metabarcoding analysis.

Genus	Counts	Percentage frequency of occurrence (%FOO)	Proportion of occurrence (POO)	Weighted proportion of occurrence (wPOO)	Relative read abundance (RRA)
*Mytilus* (Bivalvia)	89	46.5969	0.0466	0.0604	0.0639
*Monocorophium* (Malacostraca)	86	45.0262	0.0450	0.0600	0.0672
*Amathia* (Gymnolaemata)	61	31.9372	0.0320	0.0338	0.0152
*Acartia* (Copepoda)	60	31.4136	0.0314	0.0237	0.0022
*Ciona* (Ascidiacea)	54	28.2723	0.0283	0.0571	0.1214
*Amphibalanus* (Thecostraca)	47	24.6073	0.0246	0.0264	0.0677
*Caprella* (Malacostraca)	46	24.0838	0.0241	0.0144	0.0190
*Gammarus* (Malacostraca)	44	23.0366	0.0230	0.0534	0.0440
*Halacarellus* (Arachnida)	44	23.0366	0.0230	0.0202	0.0079
*Pleistacantha* (Malacostraca)	44	23.0366	0.0230	0.0257	0.0011
*Centropages* (Copepoda)	42	21.9895	0.0220	0.0155	0.0098
*Rhombognathides* (Arachnida)	38	19.8953	0.0199	0.0176	0.0024
*Crassicorophium* (Malacostraca)	35	18.3246	0.0183	0.0071	0.0041
*Harmothoe* (Polychaeta)	35	18.3246	0.0183	0.0176	0.0315
*Metridium* (Anthozoa)	35	18.3246	0.0183	0.0153	0.0583
*Lacuna* (Gastropoda)	34	17.8010	0.0178	0.0206	0.0347
*Dexamine* (Malacostraca)	32	16.7539	0.0168	0.0133	0.0082
*Halocladius* (Insecta)	32	16.7539	0.0168	0.0156	0.0483
*Modiolus* (Bivalvia)	30	15.7068	0.0157	0.0074	0.0007
*Engraulis* (Actinopterygii)	29	15.1832	0.0152	0.0133	0.0047
Total	191	‐	‐	‐	‐
Species					
*Mytilus trossulus* (Bivalvia)	66	35.2941	0.0409	0.0518	0.0730
*Monocorophium insidiosum* (Malacostraca)	64	34.2246	0.0396	0.0735	0.0618
*Acartia hudsonica* (Copepoda)	51	27.2727	0.0316	0.0188	0.0023
*Amphibalanus improvisus* (Thecostraca)	47	25.1337	0.0291	0.0320	0.0813
*Caprella mutica* (Malacostraca)	44	23.5294	0.0272	0.0226	0.0228
*Centropages hamatus* (Copepoda)	41	21.9251	0.0254	0.0217	0.0118
*Rhombognathides pascens* (Arachnida)	38	20.3209	0.0235	0.0241	0.0029
*Mytilus galloprovincialis* (Bivalvia)	37	19.7861	0.0229	0.0211	0.0037
*Amathia tertia* (Gymnolaemata)	36	19.2513	0.0223	0.0153	0.0090
*Crassicorophium bonellii* (Malacostraca)	35	18.7166	0.0217	0.0130	0.0049
*Metridium senile* (Anthozoa)	35	18.7166	0.0217	0.0200	0.0701
*Lacuna vincta* (Gastropoda)	34	18.1818	0.0211	0.0234	0.0417
*Dexamine thea* (Malacostraca)	32	17.1123	0.0198	0.0184	0.0099
*Gammarus oceanicus* (Malacostraca)	32	17.1123	0.0198	0.0496	0.0366
*Halocladius variabilis* (Insecta)	32	17.1123	0.0198	0.0192	0.0581
*Modiolus modiolus* (Bivalvia)	30	16.0428	0.0186	0.0099	0.0009
*Harmothoe imbricata* (Polychaeta)	29	15.5080	0.0180	0.0202	0.0316
*Nicolea zostericola* (Polychaeta)	29	15.5080	0.0180	0.0148	0.0051
*Membranipora membranacea* (Gymnolaemata)	26	13.9037	0.0161	0.0370	0.0064
*Ciona roulei* (Ascidiacea)	23	12.2995	0.0142	0.0096	0.0016
Total	187	‐	‐	‐	‐

*Note*: The majority of taxa identified at higher frequencies were benthic and/or sessile organisms, with many being commonly observed biofouling organisms.

When the wPOO calculations within the metabarcoding datasets were considered, certain taxa were observed at higher frequencies than would be expected based on the %FOO and POO observations. Namely, the ascidian genus *Ciona*, the amphipod genus *Gammarus* and the brittle star genus *Ophiopholis* as well as the species *Monocorophium insidiosum*, *Gammarus oceanicus* and *Ophiopholis aculeata* all had particularly large wPOO values in relation to other taxa that shared similar %FOO and POO values, suggesting that in the sample wherein these taxa were consumed, they were the predominate prey items (Tables S2–S4). Similar observations regarding the RRA were made, with certain taxa like *Ophiopholis* and *Ciona* possessing larger RRA values (0.08 and 0.12, respectively) compared to taxa with similar values of %FOO and POO (Table 1). However, RRA is not a direct proxy to detection frequency and is often influenced by primer/amplification biases, and so variation like this is not unusual or indicative of overarching phenomena.

### 3.4 Metabarcoding: Proportion of identified taxa based on total ESV and read counts

**FIGURE 4:**
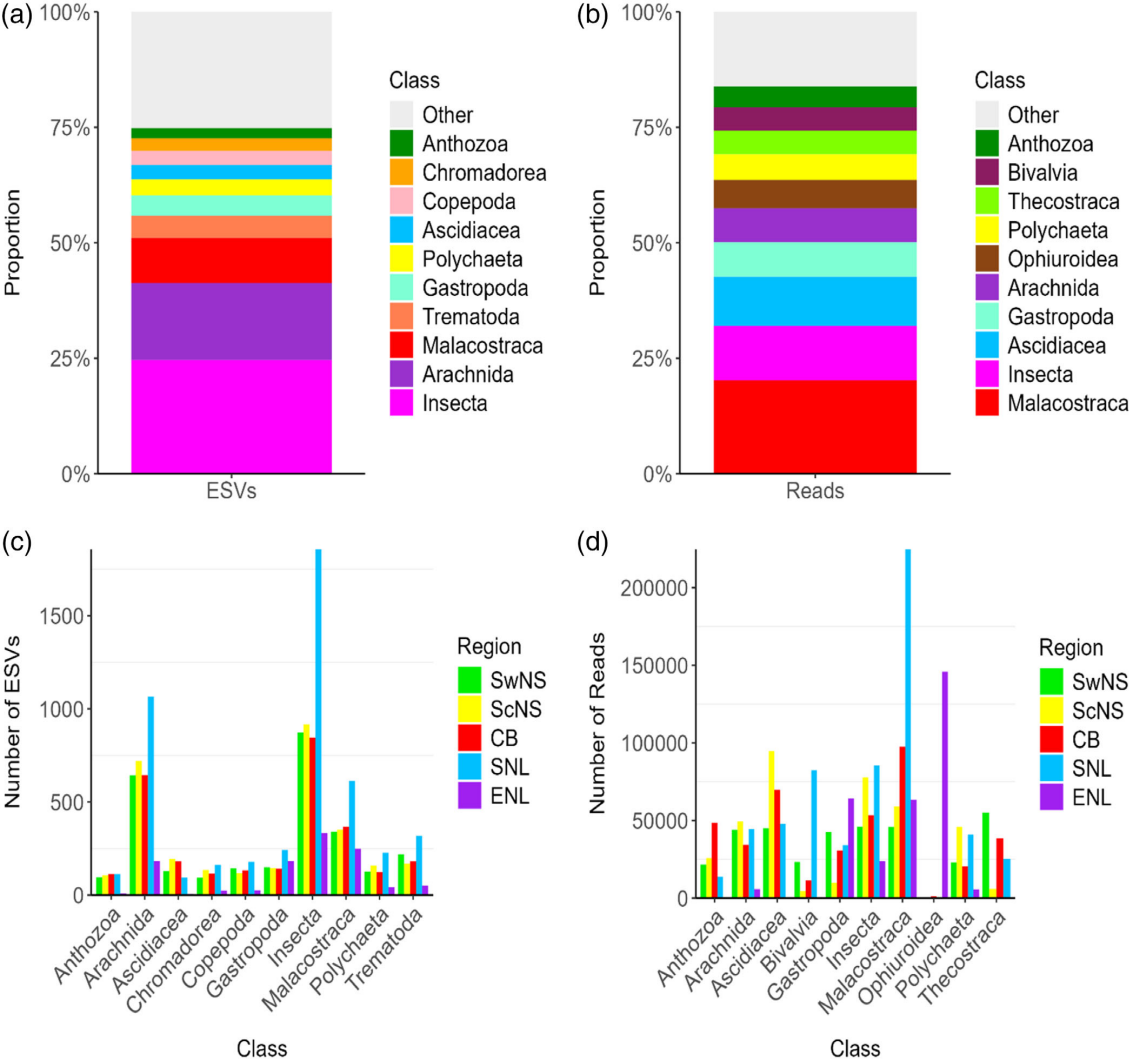
Visualizations for the entire dataset of the proportion of assigned exact sequence variants (ESVs) (a) and of total reads (b) for major taxa at the class level. Visualizations of 10 common classes for the five major sampling regions of the proportion of assigned ESVs (c) and of total reads (d).

Comparison of proportions of ESVs and of RRs for identified taxonomic groups showed that the patterns for some regions differed from those for the entire dataset. The dDNA metabarcoding further confirmed that the diet of the cunner wrasse was predominantly comprised of benthic organisms. As was observed in the morphological dataset, %ESV (Figure 4a) and RR (Figure 4b) had large proportions of identified sequences belonging to malacostracans, arachnids, gastropods and ascidians. Interestingly, two families, which were not detected in the morphological analysis, contributed to a large proportion of the ESVs (Insecta) or a large proportion of the RR counts (Ophiuroidea), respectively.

Similarly, the number of ESVs and read counts calculated for each region for the top 10 identified taxa at the class level showed that although many taxa were found commonly throughout all gut samples, certain taxa had larger numbers of either ESVs (Figure 4c) or read counts (Figure 4d) within specific regions than expected from their FOO. Regional trends of note for the ESV included larger numbers of ESVs for arachnids, malacostracans and insects in Southern Newfoundland gut samples (Figure 4c). For read counts, ascidians were characterized by high counts (>50,000) in gut samples from Southcentral Nova Scotia and Cape Breton, exceptionally high counts (>100,000) for bivalves and malacostracans in Southern Newfoundland gut samples, exceptionally high counts (>100,000) for ophiuroids in Eastern Newfoundland and very low counts (<1000) for ophiuroids in all the other sampled regions. Read counts across samples for all taxa detected by the three primer sets varied but were often high (Figure S2). However, visualizations of read counts for the top 10 detected taxa as a function of which primers were used demonstrated that the detection of certain taxa, such as *Mytilus trossulus*, required the inclusion of the mljg primer, highlighting how using multiple primers can help avoid primer bias during sequence detection (Figure S3).

### 3.5 Variation in dietary composition across geographic and temporal groups

**FIGURE 5:**
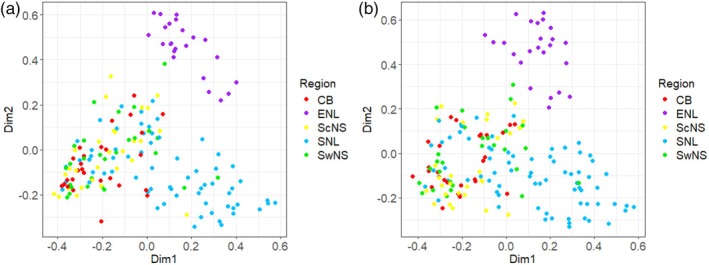
Principal coordinate analysis (PCoA) for both the species (a) and genus (b) metabarcoding datasets (presence/absence) plotted with regional colour overlays based on distance metrics calculated through the use of Sørensen's coefficient. Dimensions 1 and 2 (Dim1 and Dim2) of plot A represent 13.45% and 10.12% of the total variation, respectively, whereas dimensions 1 and 2 (Dim1 and Dim2) of plot B represent 12.66% and 9.89% of total variation.

Plots of a PCoA generated from the metabarcode datasets based on Sørensen's coefficient show that Southern Newfoundland samples appear to vary the most yet remain relatively clustered together, whereas Eastern Newfoundland samples appeared to cluster together more closely than any other region (Figure 5). In general, samples seem to cluster into two large groups dictated by geographic region, resulting in a cluster of Nova Scotian (CB, ScNS, SwNS) versus Newfoundland (ENL, SNL) samples. Further differentiation was also observed between the two regions within the Newfoundland group (Figure 5). Subsequent PERMANOVA analyses conducted to test for statistical significance of these grouping variables (Region, Location) revealed significant yet small (based on the determined F‐value) effects of region and location on dietary composition, with region having the larger proportional effect (Table S5). Another PCoA was conducted from the morphological dataset, revealing greater variance between samples in general and a lack of clear differences between regions (Figure S4). Interestingly, PERMANOVA analysis of this dataset did reveal statistically significant effects of grouping variables (Region, Location) on dietary composition (Table S5). Again, these effects were relatively minimal, with the effect of region on the morphological data in particular being smaller than the effect of region on either of the metabarcoding datasets (Table S5).

Further PCoAs were conducted based on the singular site that was sampled between the sample years to test for variance as a result of sample Year revealed slight clustering of sample years within both metabarcoding datasets that would suggest that prey selection is a function of prey availability rather than actual individual preference (Figure S5). The morphological datasets did not visually reveal any strong clustering patterns with Year (Figure S5). PERMANOVA conducted on these three datasets (Morphological, Metabarcode‐Genus and Metabarcode‐Species) confirmed statistically significant effects of the grouping variable (Year) on dietary composition within all three datasets, again with small general effect sizes (Figure S5).

Pair‐wise comparisons between all possible pairs of regions and locations were also calculated to determine where the largest differences were (Tables S7–S10). Regarding the metabarcoding data, the largest differences noted were between Eastern Newfoundland and Cape Breton (species data: *F* = 40.72, p‐adj = 0.01), Eastern Newfoundland and Southcentral Nova Scotia (species data: *F* = 33.01, p‐adj = 0.01), Eastern Newfoundland and Cape Breton (genus data: *F* = 37.01, p‐adj = 0.01) and Eastern Newfoundland and Southwest Nova Scotia (species data: *F* = 29.54, p‐adj = 0.01). The results from these analyses suggest that Eastern Newfoundland and Southern Newfoundland were the most distinct regions, with samples from Eastern Newfoundland being of particularly unique composition in relation to the rest of the data analysed.

## 4 DISCUSSION

### 4.1 Dietary composition of *T. adspersus* across Atlantic Canada

This study represents the first in‐depth investigation of *T. adspersus* dietary ecology using dDNA metabarcoding technology across a geographic range spanning 1086 km. This study also compared results from traditional morphological dietary analysis to metabarcoding using the same set of gut samples. Of the two methods, only metabarcoding routinely identified soft‐bodied taxa and often enabled resolution to the species level. We detected statistically significant dietary variation between regional, locational and temporal groupings of individuals. As hypothesized, presence and/or absence of observed prey taxa within the two groups of Newfoundland samples was the most distinct from the Nova Scotian samples, suggesting unique prey taxa were located and consumed within both study sites in Newfoundland. These results suggest that geographical separation and ecological variation in prey taxa diversity contribute to differences in observed diets between the mainland and the island of Newfoundland.

Significantly consumed prey items, in terms of composition by weight (%WC), were mussels, bryozoans, ascidians, gastropods, unidentified material and barnacles (Figure 2). However, the high relative weights of the bivalves and gastropods represented mostly indigestible shell fragments, which artificially inflated their contribution to the nutrition of this wrasse. Thus, the metric %WC, used for only the morphological dataset, was biased towards hard‐bodied prey taxa because of their longer gut residence times relative to soft‐bodied taxa. Many of the common prey taxa detected here have been previously documented in strictly morphological studies, reinforcing the consensus that *T. adspersus* is a demersal feeder preferentially consuming readily available, slow‐moving or sessile organisms (Olla et al., 1975; Patricio Ojeda & Dearborn, 1991; Whoriskey, 1983).

By contrast, dDNA metabarcoding identified soft‐bodied prey taxa such as polychaetes and sea anemones that had not been detected in the morphological analysis (Figure 3). High values of percentage FOO (%FOO) from the metabarcoding results for genera like *Mytilus* and *Ciona* were in agreement with %FOO from the morphological results for bivalves and ascidians, the respective classes for these genera (Figure 3).

Two metrics exclusive to the dDNA metabarcoding (%ESV and RRA) resulted in different rankings of prey taxa from those seen when ranking had been by FOO. Classes including Insecta, Arachnida and Malacostraca made up a high proportion of the total ESVs that were detected in the entire dataset (Figure 4a). Classes including Malacostraca, Insecta and Ascidiacea made up a high proportion of the RRA (Figure 4b). These discrepancies may result from either primer bias (i.e., the overamplification of specific taxa that are particular targets of any one chosen amplicon) or from database bias within the BOLD database itself; BOLD has an excellent coverage of many species (i.e., Canadian insects) which are therefore more frequently assigned to the genus or species level (Porter et al. 2019), than are Canadian marine taxa which are comparatively poorly covered (Schultz & Hebert, 2024).

Some of the prey species found in this study were frequently detected in a recent study of natural diet variation in wild populations of two of the five commonly used cleaner wrasses within European salmon aquaculture (Bourlat et al., 2021). Metabarcoding of gut contents of wild‐caught goldsinny (*C. rupestris*) and corkwing (*S. melops*) wrasse collected from three sites across a 12‐km marine protected area in southwestern Sweden found large proportions of ASVs (amplicon sequence variants, analogous to the ESVs in the current study) were identified as arthropods in the diet of both species (69% for *C. rupestris* and 97% for *S. melops*). A large percentages of these arthropods belonged to the class Malacostraca (51% for *C. rupestris* and 90% for *S. melops*; Bourlat et al., 2021). Malacostracan ESVs were not as common in the current study on *T. adspersus* yet represented the third most frequently detected group at the class level, composing 9.81% of all detected ESVs (Table S11). A solely morphological study on European cleaner wrasse diets found that common prey items among ballan wrasse (*L. bergylta*) dietary samples included various hard‐shelled species like decapods and bivalves (Deady & Fives, 1995). Commonalities in the diet of *T. adspersus* and the diet of the European wrasses used as cleaner fish options highlight a shared ecological niche and dietary breadth, suggesting, based on the success of cleaner wrasses in Europe to combat this issue, the potential for success in the event that *T. adspersus* is employed in a similar manner in the Northwestern Atlantic.

In the current study, the salmon louse (*L. salmonis*) was uniquely detected in three gut samples (%FOO >1.5%) from three different sites in the metabarcoding dataset specifically, which would not have been detected had only a morphological approach to dietary analysis been employed (Table S4). dDNA metabarcoding detected the salmon louse (*L. salmonis*) inside the ethanol‐preserved stomachs of lumpfish (*C. lumpus*) living within sea cages containing Atlantic salmon (*S. salar*) that were frequently heavily infected with lice, but careful morphological analyses of the same sets of stomachs found no evidence of lice (Roy & Boulding, 2024). The low %FOO of *L. salmonis* for the morphological analyses within this *C. lumpus* study is likely a result of crustaceans such as *L. salmonis* possessing a thinner cuticle, which can be digested at a faster rate than other crustaceans with thicker cuticles such as krill (Roy & Boulding, 2024). *L. salmonis* placed in *C. lumpus* stomachs with tubing had a mean digestion time of 15 h at 9°C (Staven et al., 2021), and the digestion rate would likely be even quicker inside a stomach‐less (agastric) species of wrasse (Le et al., 2019), such as *T. adspersus*. This demonstrates that, for taxa with soft body parts, or taxa that possess thinner or frail hard parts that quickly breakdown during digestion, the use of metabarcoding is invaluable as a detection technique. This is especially true when the detection of specific frail taxa (such as problematic species such as the salmon louse within sea cage environments) is of particular interest.

Many of the prey in this study are widely considered pest biofouling species, supporting the integration of *T. adspersus* as a potential cleaner fish in marine sea cages. The genus *Ciona* was one of the most frequently identified prey items, especially in more southern samples, and is of particular interest for aquaculture managers in more northern waters given its recent invasion of the area (Deibel et al., 2014; Ma et al., 2017; McKenzie et al., 2015; Therriault & Herborg, 2008). The large contribution of ascidian sequences (Figure 4b), as well as the frequent detection of *Ciona* sequences in different samples (Figures 3b and S6), implies a significant contribution of this group to overall dietary composition of *T. adspersus*, especially for the individuals from southern regions (Figure 4c,d). This suggests potential for *T. adspersus* to act as a direct method for prevention of the colonization of this invasive species on sea cages.

### 4.2 Regional variation in dietary makeup

Results from the PCoA analysis and subsequent PERMANOVAs revealed various patterns in prey consumption that varied with region, location and year. The primary relationship of note in the dDNA metabarcoding dataset is the clustering of samples at the regional level into three main subgroups with small amounts of admixture (on the basis of diet similarity as determined by prey species occurrences). This occurred across both taxonomic levels investigated (genus and species) and corresponds to samples grouped by region into Eastern Newfoundland samples, Southern Newfoundland samples and Nova Scotian samples. This would suggest that there is some form of geographic and temporal influence on the dietary composition of a given *T. adspersus* throughout Atlantic Canada. Eastern Newfoundland in particular was the most unique compared to all other regions, and these results match our anticipated results based on the initial hypothesis of a faunal break caused by isolation by distance and ecological constraints imparted by the Gulf of St. Lawrence which separates these groups (Dorant et al., 2022; Moir, 2021). The Gulf of St. Lawrence has been observed to impart variation in the population genetics of species like the American lobster (*Homarus americanus*) that occur both to the north and south of the gulf (Dorant et al., 2022), but this is the first study of dietary trends in an invertebrate‐consuming fish species within Atlantic Canada that demonstrated significant differences in dietary composition across the entire region. Another study found congruent population genetic structure in the Atlantic cod (*Gadus morhua*), the American lobster (*Homarus americanus*), the northern shrimp (*Pandalus borealis*) and the sea scallop (*Placopecten magellanicus*), consisting of a genetic break along the coast of Nova Scotia at a latitude around 44.6° N which was argued to be the result of current boundaries and minimum winter temperature gradients (Stanley et al., 2018). Similarly, strong environmental gradients imparted by the Gulf of St. Lawrence on benthic invertebrate community composition (Dorant et al., 2022; Stanley et al., 2018) may directly influence dietary composition of *T. adspersus* to the north compared to the south of the Gulf itself (Moir, 2021). However, the PCoAs from the morphological diet datasets did not reveal these trends, likely driven by the smaller amount of prey categories identified.

Some prey species have range limits that coincide with observed faunal breaks in our cunner diet datasets. The invasive ascidian *Ciona* has gradually become well established in Nova Scotia, but only recently has established fragmented populations along the southern shores of Newfoundland (Deibel et al., 2014; LeGresley et al., 2008; McKenzie et al., 2015; Sephton et al., 2011). The %ESVs and %RRA of *Ciona* were relatively high in Nova Scotian gut samples but absent in Eastern Newfoundland gut samples (Figure 4c,d).

In several cases, regional differences in consumption frequencies of a particular prey taxon cannot be explained by geographical range limits of that prey. Many species, including various amphipods like *Gammarus oceanicus* and *Gammarus lawrencianus*, the polar shrimp *Lebbeus polaris*, the gastropod *Lacuna vincta* and the brittle star *Ophiopholis aculeata* were frequently detected in Newfoundland cunner gut samples, yet were rarely consumed in Nova Scotia samples, despite their largely ubiquitous geographical ranges that overlap the entirety of the study region (WoRMS, 2024a, 2024b, 2024c, 2024d, 2024e). Similarly other largely ubiquitous species such as the bay barnacle *Amphibalanus improvisus* and the frilled anemone *Metridium senile* were found in substantially larger proportions in Nova Scotian gut samples compared to Newfoundland gut samples (WoRMS, 2024f, 2024g). Despite many of these species occurring across the entirety of the study area, many of them were consumed only in select regions (Figure 4c,d, WoRMS, 2024e, 2024f, 2024g) and as such greatly contributed to observed differences between regions (Figure 5a,b). These differences could be the result of prey preference varying across geographic locations because of population‐level genetic divergence in preferred prey or could be a function of local prey availability at a given sampling location at a particular time. However, because our temporal comparisons at a shared site also reveal differences in dietary composition, it is also possible that abundances and availability of prey, despite most ecological conditions remaining relatively constant, varied across the sampling periods. This just as well could have led to the observed differences between sample years, and as a result, complicates any conclusions drawn based on the dietary preference. These issues could also contribute to divergences in observed dietary composition at different sites sampled in different years [i.e., Eastern Newfoundland (2022) compared to the Nova Scotian (2018) samples], again complicating potential takeaways regarding dietary divergence between regions.

Both the morphological and metabarcoding diet datasets showed significant temporal differences in prey composition at the single site (Hermitage Bay, NL) that was sampled in 2018 and again in 2022 (Table S6). Temporal variation, and more specifically seasonal prey fluctuation, has been demonstrated to influence dietary composition in fish species (Eriksen et al., 2021; Facendola & Scharf, 2012). This phenomenon could be influencing the results seen between years at the Hermitage Bay site. This would, if prey selection is purely a function of availability rather than electivity of the predator, result in differences in dietary composition between the sample years, which was observed in this study. However, although samples were collected around the same time of year and thus ecological conditions were expected to be constant across sampling seasons, there is always the possibility that community assemblages of invertebrates vary from year to year even when environmental conditions remain similar. Parsing out dietary composition as a function of availability versus electivity based on these data alone is therefore not reliable. There remains a need for the simultaneous investigation of benthic invertebrate community composition and dietary composition of the cunner to more concretely determine this relationship.

Previous studies on dietary selection in fish species have revealed trends between electivity (intentional prey choice favouring specific taxa/groups) and specific functional traits (morphological and/or behavioural characteristics) of either the predator or prey species (Rodriguez‐Lozano et al., 2016; Ludwig et al., 2024). Based on the observations of differentiation in *T. adspersus* dietary makeup between sample sites, as well as reports of electivity in prey consumption for various fish species within the literature, there exists a need for the simultaneous study of site‐specific benthic‐community composition and functional trait observations of both predators and prey (Ludwig et al., 2024; Rodriguez‐Lozano et al., 2016), alongside analyses of *T. adspersus* dietary makeup. Further work could distinguish whether *T. adspersus* predictably selects prey (electivity), or whether its consumption of a given prey item is based more on the energetics of prey capture per unit time (i.e., optimal foraging theory based on prey abundances; Pyke et al., 1977).

### 4.3 Morphological versus metabarcoding diet analysis: Insights

Discrepancies in the results between the initial morphological analysis and the subsequent DNA metabarcoding analysis highlight a common limitation of morphological analysis. Although the information from the morphological analysis was useful, the obvious limitations to the accuracy of taxonomic identification that result from this methodology highlight the need for metabarcoding as a means of generating the most accurate picture of dietary composition possible. Initially, the identification of around 11 different taxonomic groups was derived from the morphological study, with the finest level of classification possible being the class level of certain groups like bivalves. This is in comparison with more than 200 identified species using the highly conservative criterion of 80% shared base pairs within the dDNA metabarcoding datasets, which represented a large increase in the frequency and specificity of prey species identification. Various sources of error that occurred during morphological gut analysis are likely responsible for this. This includes simple human identification errors of cryptic taxa, to the state of digestion of prey items which, in an agastric species like *T. adspersus*, can be high given their short gut‐transit times (Hayes & Volkoff, 2014; Le et al., 2019). This is not to say that the morphological analyses are not without merit on their own. Estimations for the proportion of which a given prey item contributes to the total amount of prey consumed based on dDNA metabarcoding data can be erroneous. This is typically a result of primer/recovery biases of certain taxa sequences (Deagle et al., 2019). Thus, this study acts to solidify the need for both forms of analysis in unison to generate the most holistic image of dietary makeup for a given species.

Recent studies investigating the dietary composition of fish species often employ both morphological methods and dDNA metabarcoding (Günther et al., 2021; Maes et al., 2022; Takahashi et al., 2020). Morphological methods can be more quantitative and can help assess whether prey items detected by metabarcoding are due to genuine predator–prey interactions or are the result of accidental consumption (Günther et al., 2021). For example, in a study investigating Atlantic bluefin tuna (*Thunnus thynnus*) diets, low levels of detection (as RRA) of various taxa such as annelids, platyhelminths and xenacoelomorphs in metabarcoding analyses were attributed to accidental consumption, as it is unlikely that very small invertebrates would be targets of predation by *T. thynnus* (Günther et al., 2021). In the pre‐filtered datasets of our *T. adspersus* diet study, small‐bodied taxa like Xenacoelomorpha were detected at low levels of RRA, suggesting similar accidental consumption or environmental contamination. Another study on the dietary composition of polar cod (*Boreogadus saida*) using both metabarcoding and morphological analysis also concluded that dDNA metabarcoding tools cannot distinguish primary prey from secondary (accidental) prey items (Maes et al., 2022).

### 4.4 Implications and future work

This in‐depth investigation of the dietary composition of the cunner wrasse (*T. adspersus*) in Atlantic Canada assesses its potential as a facultative cleaner fish species in Atlantic salmon (*S. salar*) aquaculture through analysis of its natural diet. This study details the significant overlap between species that are frequent contributors to biofouling communities in aquaculture and species that are notable constituents of the diet of the cunner. This includes bivalves such as those belonging to the genera *Mytilus* and *Modiolus*, invasive tunicates in the genus *Ciona*, as well as other common biofouling organisms such as thecostracan barnacles. This study highlights that these similarities may beget cleaning behaviour in the cunner, if it is integrated as a cleaner fish in sea cage environments. The significant amount of overlap between species considered as pests in aquaculture and species acting as major dietary constituents for the cunner could, if cunner are stocked in sea cages, leads to reductions in the rate of development of biofouling communities through direct predation by the cunner.

Preliminary observations suggested that the dietary composition of *T. adspersus* should vary between Nova Scotian samples and Newfoundland samples, driven by a faunal break imparted via geographic separation by the Gulf of St. Lawrence. The results of this study indicate that differences in diet between Nova Scotia and Newfoundland can be partially explained by geographic range limits imposed on certain common prey taxa, such as the invasive ascidians in the genus *Ciona*. However, explanations for observed regional differences in the consumption of more ubiquitously distributed taxa, such as the sea anemones in the genus *Metridium*, are less apparent and require further investigation.

A study of trends in prey consumption by *T. adspersus* as a function of benthic diversity at a given sample site/time needs to be further investigated, as prey selection based on ecological phenomena such as optimal foraging may occur but could not be ruled out by this gut analysis study. Further, investigation of functional trait variation in both predator (*T. adspersus*) and consumed prey species as a function of prey abundance could determine whether true dietary electivity is occurring in this system. There also exists a need for trials of *T. adspersus* within aquaculture sea cages to determine the effectiveness of this species as a cleaner fish of biofouling animals in controlled environments. There remain further avenues for controlled dietary and physiological experiments regarding *T. adspersus* feeding ecology within sea cage environments, as well as a need for the investigation of the potential of this species to be reared as broodstock if it is to be stocked in aquaculture pens as a biological pest control solution.

## AUTHOR CONTRIBUTIONS

Christopher J. D. Bender: Conceptualization, data curation, formal analysis, investigation, methodology, visualization, writing – original draft, writing – review and editing. Camden D. Moir: conceptualization, data curation, formal analysis, investigation, methodology, software, validation, writing – review and editing. Mehrdad Hajibabaei: methodology, software, resources. Elizabeth G. Boulding: conceptualization, funding acquisition, methodology, project administration, resources, supervision, writing – review and editing.

## FUNDING INFORMATION

Natural Sciences and Engineering Research Council of Canada (NSERC) Strategic Partnership grant with Cooke Aquaculture Inc. (CAI) to E. G. Boulding (P. I.) and L. R. Schaeffer (co‐P. I.), NSERC Discovery grants awarded to E. G. Boulding, and the work‐study programme at the University of Guelph. M. Hajibabaei's laboratory was supported by the Government of Canada through Ontario Genomics, Genome Canada and Environment and Climate Change Canada.

## CONFLICT OF INTEREST STATEMENT

The authors declare there are no competing interests.

## Supporting information


**Data S1.** Supporting information.

**Data S2.** Supplementary figures.

**Data S3.** Supplementary tables.

## Data Availability

Data generated during this study are provided in the published article and its Supplementary Materials. After acceptance, raw reads will be submitted to NCBI, and FASTA files of the ESVs will be available as Supplementary Materials. Reference sequences used to train classifier will be available on BOLD or GenBank. Bioinformatics pipeline used for metabarcoding assignments is available from the MetaWorks Github located here: https://github.com/terrimporter/MetaWorks.
