# Supplementary material for: Morphological and metabarcoding dietary analysis of the cunner wrasse ( Tautogolabrus adspersus ) revealed significant regional variation, with large overlap between its common prey species and biofouling animals living on salmonid sea cages
Source: J Fish Biol. 2025 Mar 4;107(1):143–60. doi: 10.1111/jfb.70013 (PMC12327174; doi:10.1111/jfb.70013)
Supplement: Supplementary file 1 — Data S1. Supporting information. [file JFB-107-143-s002.docx]

**Supplementary Methods**

**Bioinformatics: Taxonomic Assignments and Data Filtration**

Raw sequence pairing utilized SeqPrep v1.3.2 (St. John 2016), sequence primers were trimmed with CUTADAPT v2.6 (Martin 2011), and samples were combined for global analysis using VSEARCH v2.14.1 (Rognes et al. 2016). Quality filtering of trimmed sequences included the removal of reads with: 1) lengths less than 150 bp, 2) Phred scores less than 20 at read ends, and 3) a Ns count greater than 3 per sequence. Dereplication of data was preformed to preserve only novel reads using the ‘derep_fulllength’ setting within VSEARCH. The‘unoise3’ algorithm (Edgar 2016) was used simultaneously with ‘uchime3_denovo’ within VSEARCH to denoise reads, which included the removal of sequencing errors, all PhiX contaminated reads as well as putative chimeric sequences, and rare singleton or doubleton sequences with fewer than 3 identical reads. Using the ‘unoise3’ algorithm in VSEARCH, reads were organized into zero-radius operational taxonomic units (OTUs). Assignments of taxonomic identity to generated ESVs was completed through the use of the naïve Bayesian COI Classifier v5.1.0 (Porter and Hajibabaei 2018). This classifier was designed for taxonomic identity assignments based on statistical probability at all major levels of classification using k-mer frequencies (sequential k-mers of 8 bp in length) as well as Bayesian assessment of ESV query sequences (Porter and Hajibabaei 2022). Pseudogene filtering was also applied during runs of classification through the MetaWorks pipeline (Porter and Hajibabaei 2021). COI reference sequences were obtained from GenBank (prior to April 2019) and the BOLD database’s BIN system (data release: iBOL_phase2.0_COI.tsv to iBOL_phase_6.50_COI.tsv) and subsequently incorporated within this classifier. Precise accuracies of the BIN system may help illuminate species differentiation through the use of multiple statistical clustering methods, allowing for the detection of species presences beyond traditional recordings of Linnean descriptions. However, the BIN system might also be prone to improper grouping of more ambiguous taxa, such as the phylum Cnidaria.

A taxonomy matrix of ESVs in the form of a .csv file was produced by the MetaWorks v1.12 pipeline, which was reformatted using R v4.3.1 within RStudio v12.1.402 (RStudio 2024) through the use of various custom scripts that are publicly available at <https://github-com/Hajibabaei-Lab/HajibabaeiEtAl2019>. Metazoan DNA sequences were primarily considered as potential prey items, as microbes, phytoplankton, and macroalgae were not direct targets of amplification and may represent various host symbionts and/or parasites. Potential diet contaminants resulting from the bait used in collections had their sequences removed from the taxonomy matrix using the ‘grep’ command. Removed taxa included the family of wrasses to which the *T. adspersus* belongs (‘Labridae’), and known taxa that had been used as bait, such as turkey (*Melagris gallopavo*), chicken (*Gallus gallus*), pork (*Sus* *domesticus*), beef (*Bos taurus*) and herring (*Clupea* spp.). Only high confidence taxonomic assignments were retained at the finest levels of classifications, in which bootstrap cutoffs of ≥ 0.80, ≥ 0.30, and ≥ 0.10 for shared base pairs at the species level (sBP) were applied for analysis at the species, genus, and family level, respectively. This was done to retain assignments with a 95-99% confidence of identity (CI), as such filtration levels are recommended by Porter and Hajibabaei (2018) to ensure query sequences of ~200 bp are correctly assigned using the COI Classifier. Varying bootstrap cutoffs resulted in differing sizes of datasets at specific taxonomic resolutions, namely a reduction in total sample size in datasets filtered at the species level, from the initial N= 191 to N= 187 (Species). Control samples were removed from the ESV matrix for downstream analysis.

**References:**

Edgar, R.C. 2016. UNOISE2: improved error-correction for Illumina 16S and ITS amplicon sequencing. bioRxiv: 081257. Cold Spring Harbor Laboratory. doi:10.1101/081257.

Martin, M. 2011. Cutadapt removes adapter sequences from high-throughput sequencing reads. EMBnet J. **17**(1): 10. doi:10.14806/EJ.17.1.200.

Porter, T.M., and Hajibabaei, M. 2018. Scaling up: A guide to high-throughput genomic approaches for biodiversity analysis. Mol. Ecol. 27(2): 313–338. doi:10.1111/MEC.14478.

Porter, T.M., and Hajibabaei, M. 2021. Profile hidden Markov model sequence analysis can help remove putative pseudogenes from DNA barcoding and metabarcoding datasets. BMC Bioinform. 22: 256. doi:10.1186/s12859-021-04180-x

Rognes, T., Flouri, T., Nichols, B., Quince, C., and Mahé, F. 2016. VSEARCH: A versatile open source tool for metagenomics. PeerJ. 2016(10). doi:10.7717/PEERJ.2584.

RStudio. 2024. RStudio | Open source & professional software for data science teams - RStudio. Available from <https://www.rstudio.com/>.

St. John, J. 2016. GitHub - jstjohn/SeqPrep: Tool for stripping adaptors and/or merging paired reads with overlap into single reads. Available from <https://github.com/jstjohn/SeqPrep>.
