# Supplementary material for: Morphological and metabarcoding dietary analysis of the cunner wrasse ( Tautogolabrus adspersus ) revealed significant regional variation, with large overlap between its common prey species and biofouling animals living on salmonid sea cages
Source: J Fish Biol. 2025 Mar 4;107(1):143–60. doi: 10.1111/jfb.70013 (PMC12327174; doi:10.1111/jfb.70013)
Supplement: Supplementary file 2 — Data S2. Supplementary figures. [file JFB-107-143-s001.pptx]

## Slide 1
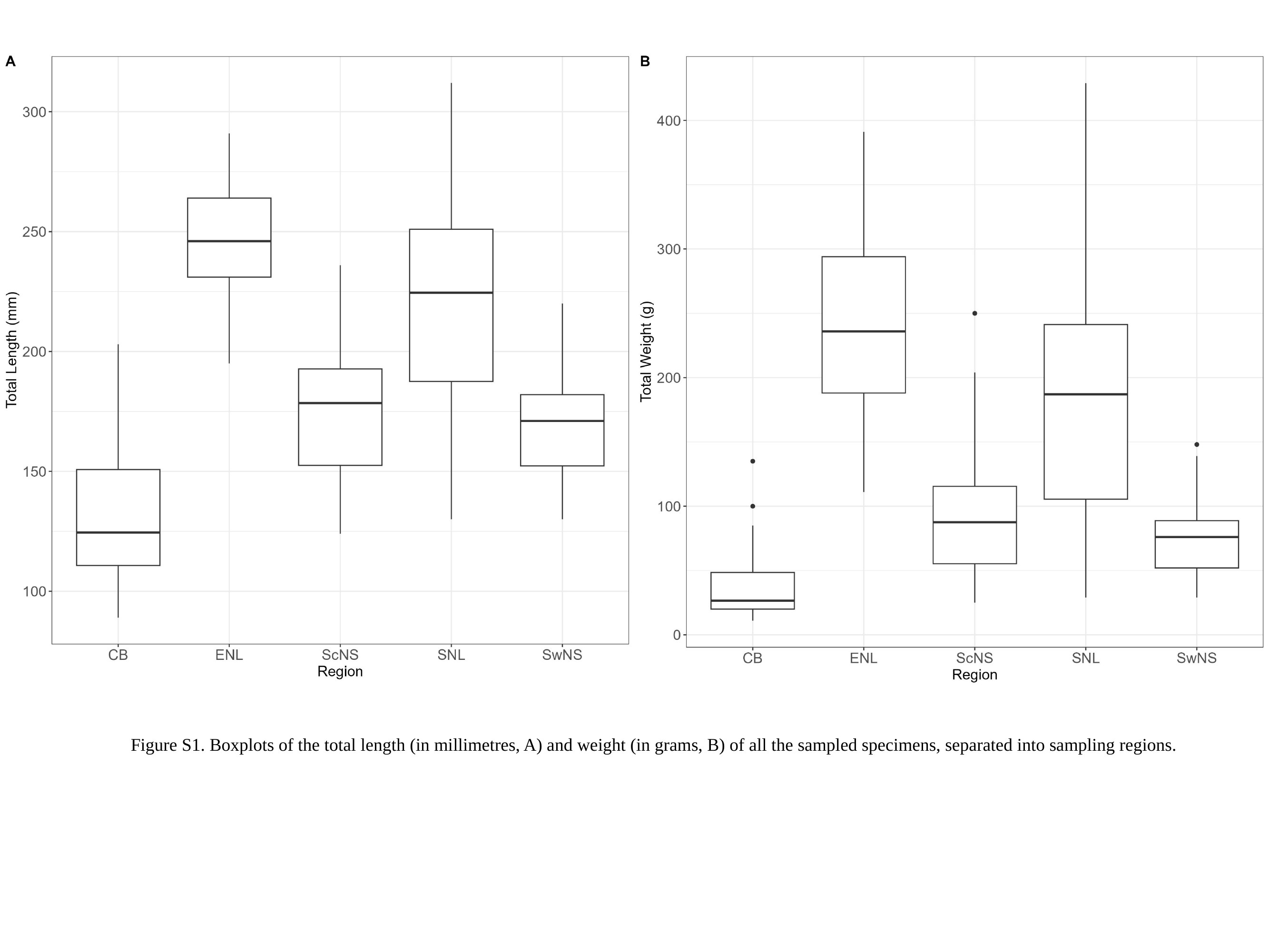

Figure S1. Boxplots of the total length (in millimetres, A) and weight (in grams, B) of all the sampled specimens, separated into sampling regions.

## Slide 2
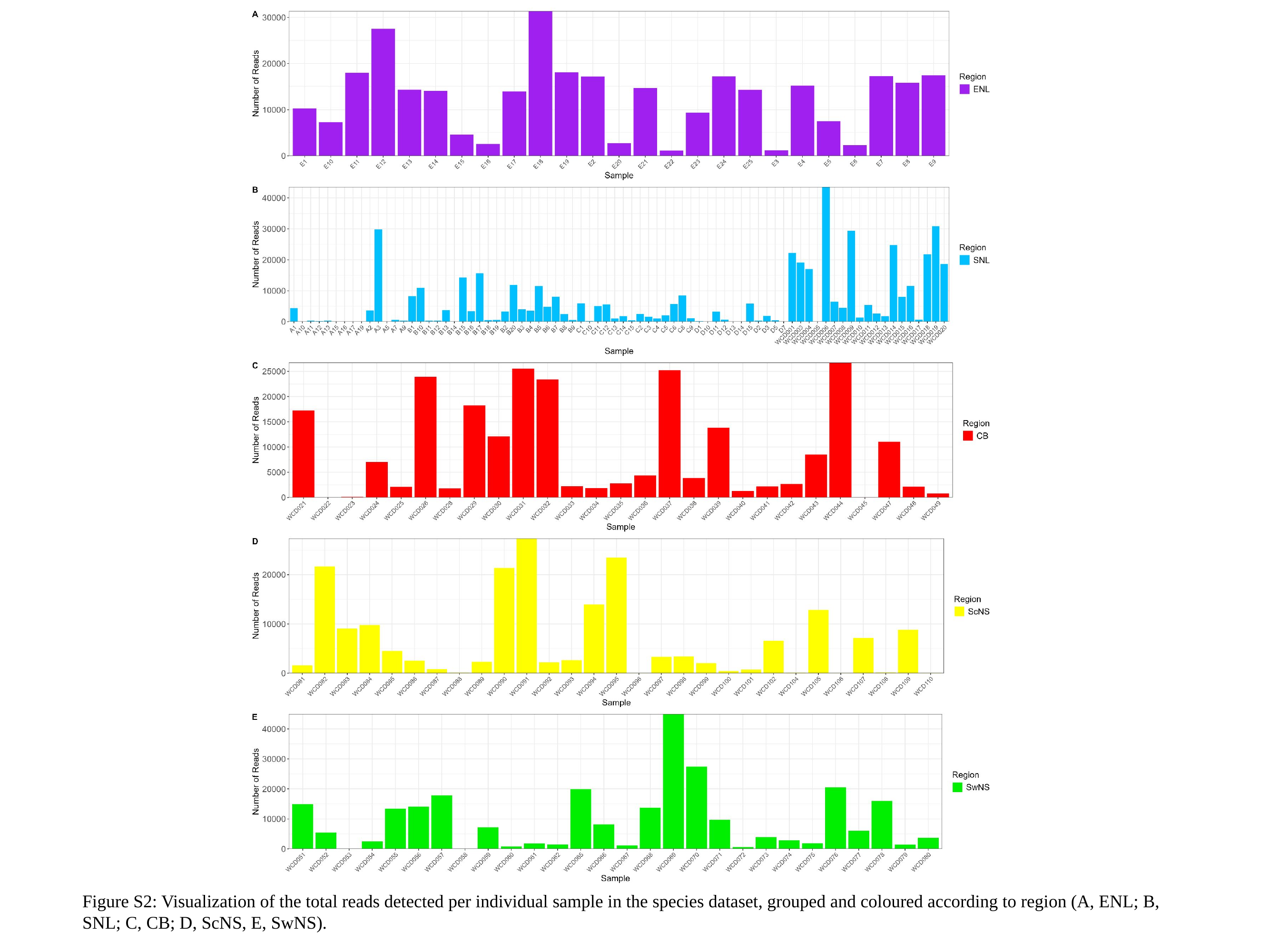

Figure S2: Visualization of the total reads detected per individual sample in the species dataset, grouped and coloured according to region (A, ENL; B, SNL; C, CB; D, ScNS, E, SwNS).

## Slide 3
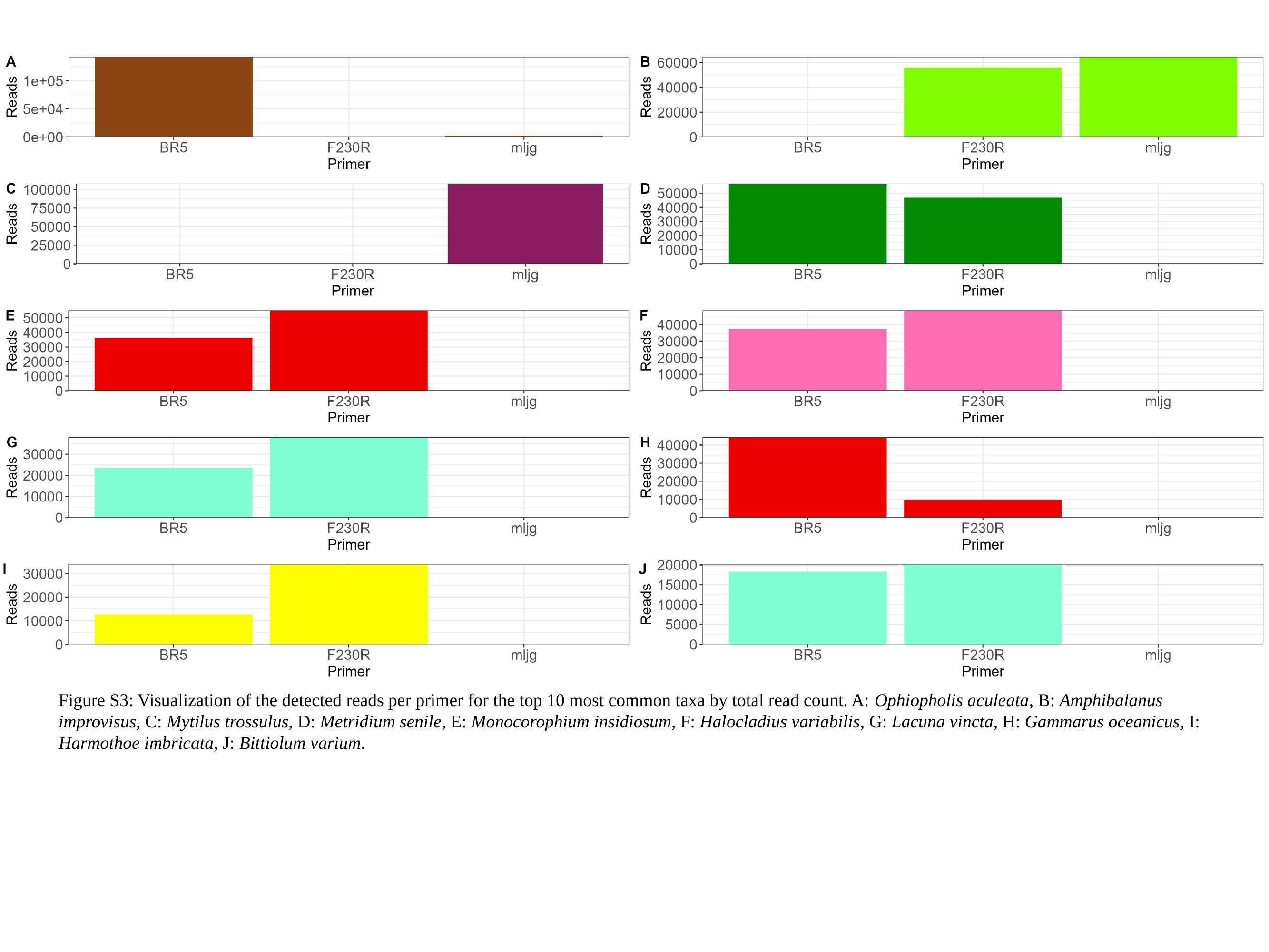

Figure S3: Visualization of the detected reads per primer for the top 10 most common taxa by total read count. A: Ophiopholis aculeata, B: Amphibalanus improvisus, C: Mytilus trossulus, D: Metridium senile, E: Monocorophium insidiosum, F: Halocladius variabilis, G: Lacuna vincta, H: Gammarus oceanicus, I: Harmothoe imbricata, J: Bittiolum varium.

## Slide 4
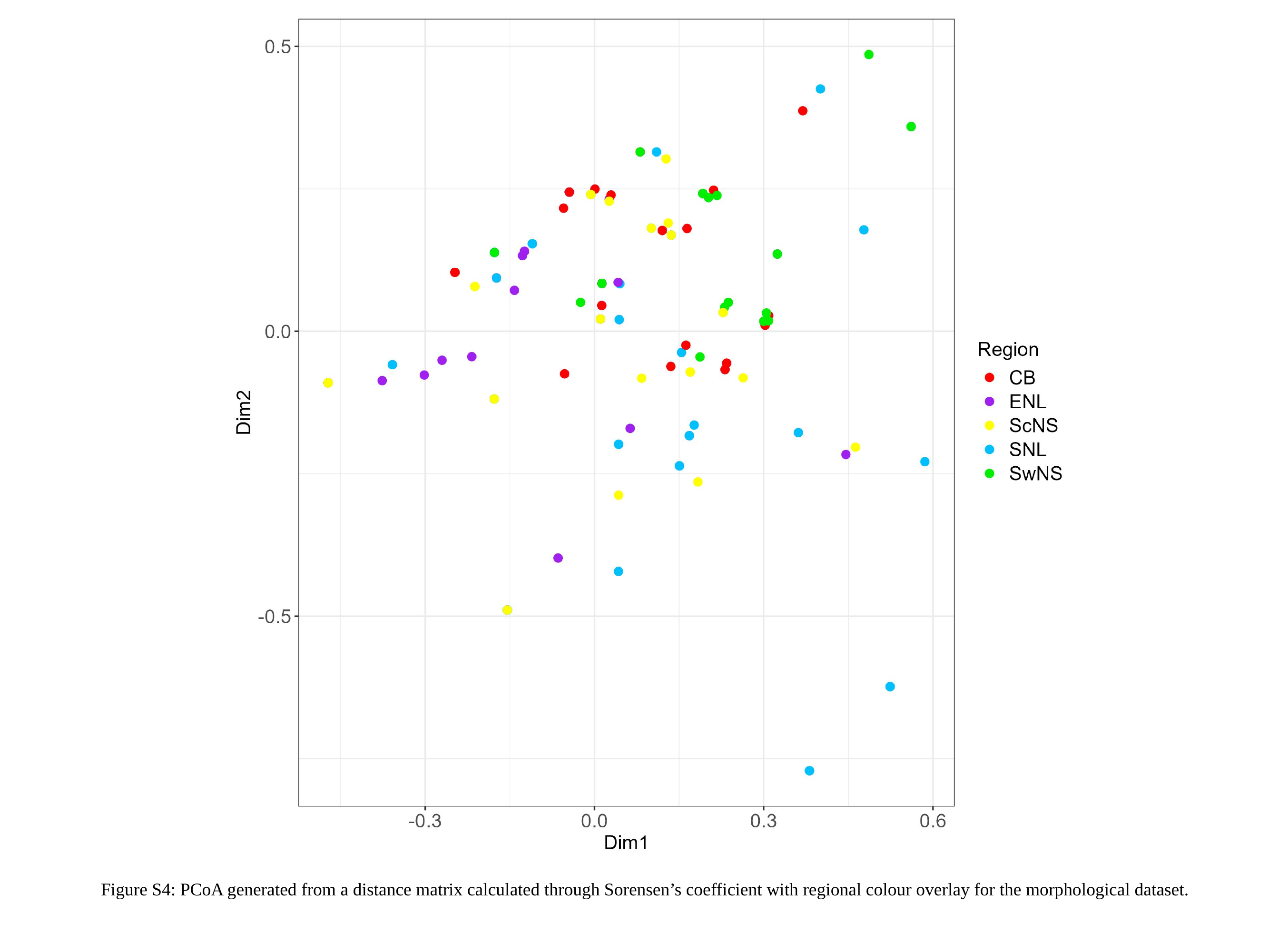

Figure S4: PCoA generated from a distance matrix calculated through Sorensen’s coefficient with regional colour overlay for the morphological dataset.

## Slide 5
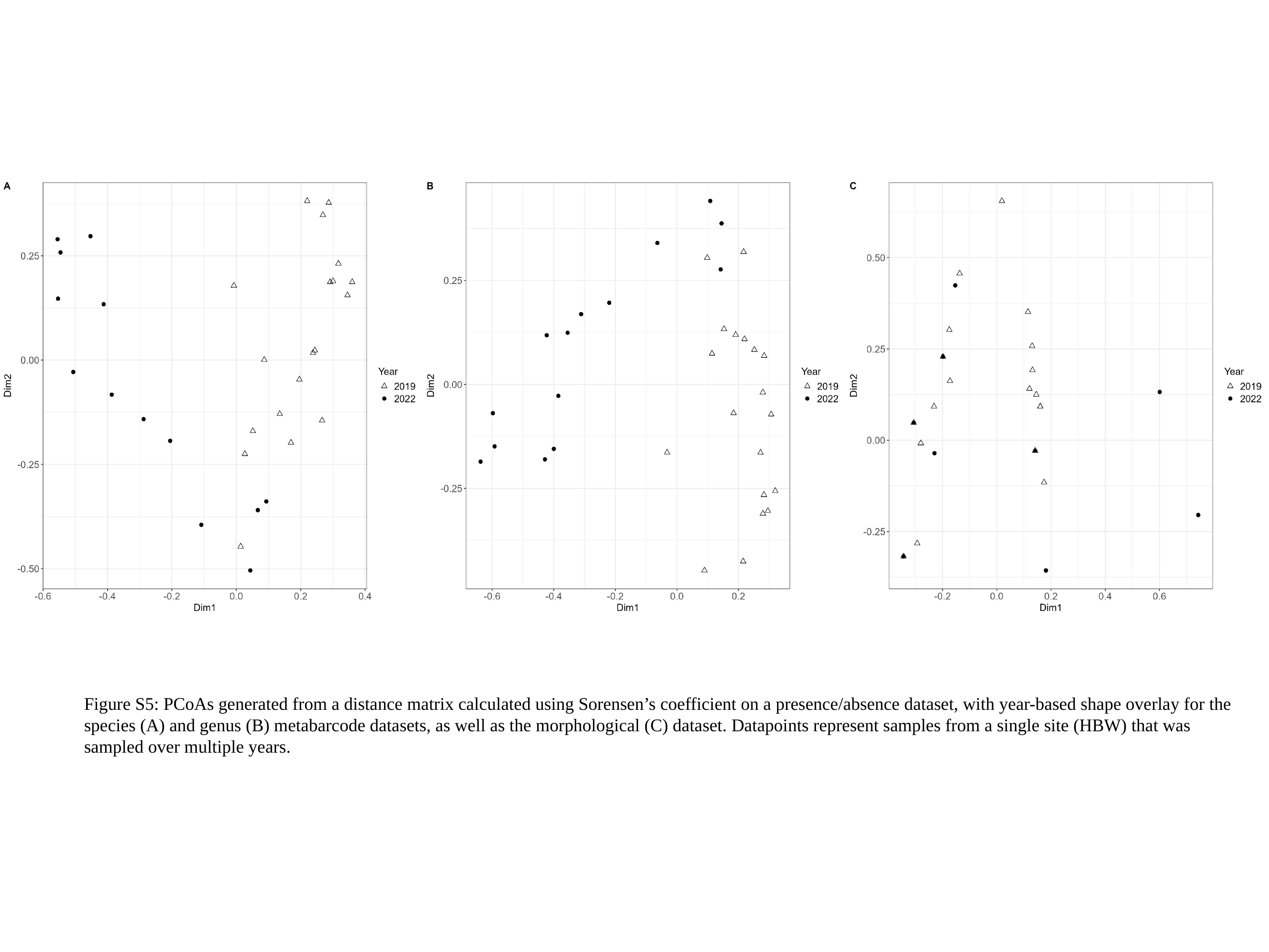

Figure S5: PCoAs generated from a distance matrix calculated using Sorensen’s coefficient on a presence/absence dataset, with year-based shape overlay for the species (A) and genus (B) metabarcode datasets, as well as the morphological (C) dataset. Datapoints represent samples from a single site (HBW) that was sampled over multiple years.

## Slide 6
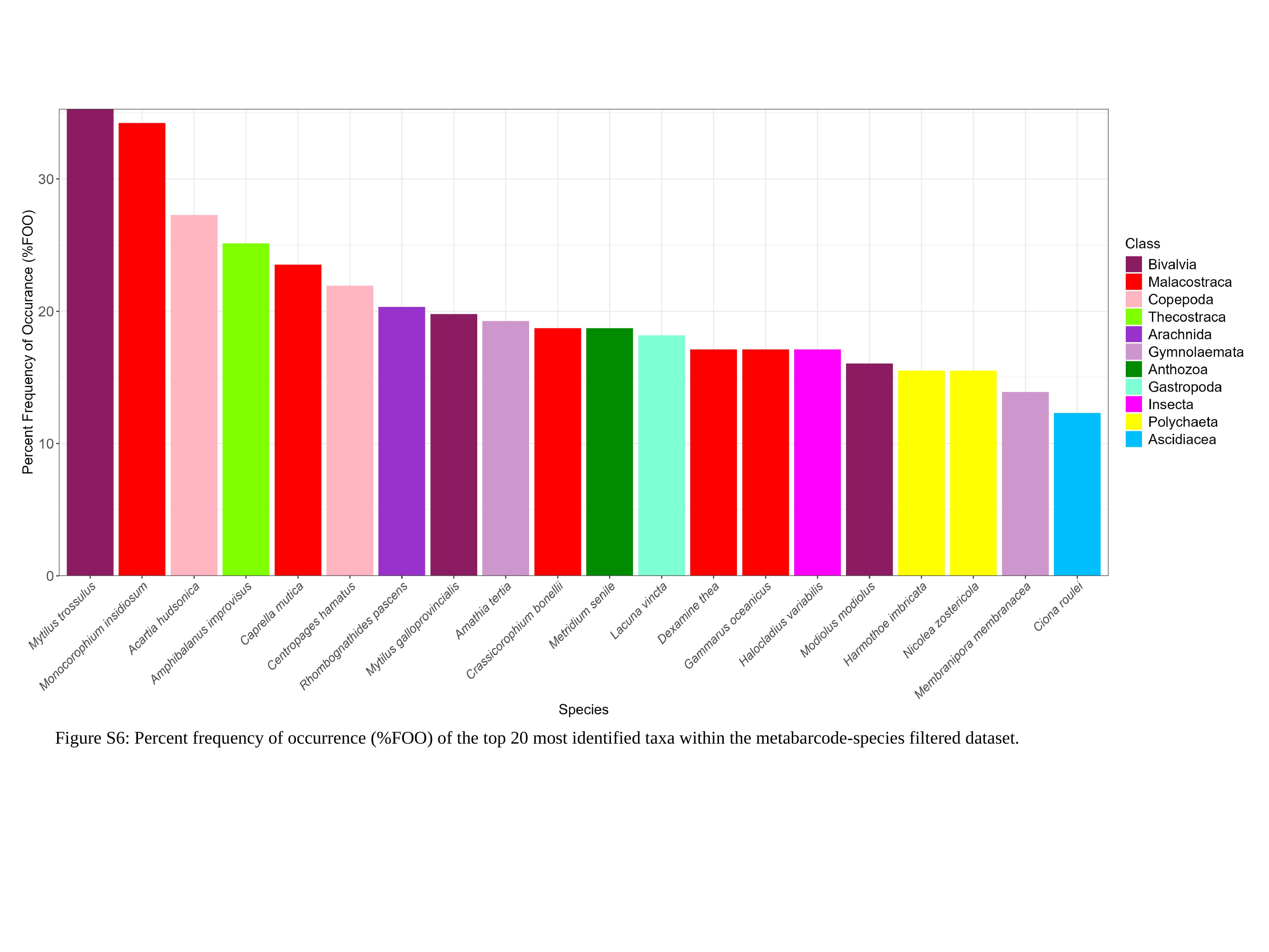

Figure S6: Percent frequency of occurrence (%FOO) of the top 20 most identified taxa within the metabarcode-species filtered dataset.
